# Supplementary material for: Content-rich biological network constructed by mining PubMed abstracts
Source: BMC Bioinformatics. 2004 Oct 8;5:147. doi: 10.1186/1471-2105-5-147 (PMC528731; doi:10.1186/1471-2105-5-147)
Supplement: Additional File 5 — The original Chilibot query results of the term "long-term potentiation (LTP)" and 22 other terms, limiting the latest references analyzed to the years 1990, 1995, 2000, and 2004. [file 1471-2105-5-147-S5.bz2 › chilibotAdditionalFile5/ltp2000/html/NMDA_ZIF268.html]

 


 **NMDA** and **ZIF268** 
  
Found 14 abstracts in PubMed,  **14 abstracts were retrieved and analyzed**.  


---

 Search Google  |
 PDF files only 
|  EDU domain only 

---

**Interactive relationship** (e.g. stimulation, inhibition, etc)

- Double immunostaining revealed that many of the constitutively expressed  **Zif268**  positive neurons were GABAergic but very few were cholinergic or neuronal nitric oxide synthase nNOS positive, and some of the  **Zif268**  positive neurons were also immunopositive for a glutamate  **NMDA**  receptor subunit NR1 or NR2A.  Ref: 10712658 Eur J Neurosci, 2000
- CPP, a competitive  **NMDA**  antagonist, showed a similar pattern of effects on cfos and  **zif268**  mRNA expression.  Ref: 9552172 Synapse, 1998
- Increased striatal  **zif268**  was dependent mainly on DA D1 and to a lesser extent on non  **NMDA**  glutamate receptors and was not altered by inhibition of nitric oxide synthase NOS.  Ref: 9313891 Brain Res, 1997
- These results suggest that immediate early gene  **zif268**  may, in part, play a role in the neurotoxicity of  **NMDA**  receptor antagonists such as PCP.  Ref: 10482811 Brain Res, 1999
- D1 dopamine receptor mediated induction of  **zif268**  and c fos in the dopamine depleted striatum differential regulation and independence from  **NMDA**  receptors.  Ref: 8708002 J Comp Neurol, 1996

**Parallel relationship** (e.g. studied together, co-existance, homology, etc.)

- Like the competitive antagonists, intrastriatal infusion of the non competitive  **NMDA**  antagonist MK 801 partially decreased c fos... mRNA in the area around the microdialysis probe.  Ref: 8708002 J Comp Neurol, 1996
- The combined administration of IBMX and eticlopride induced gene expression that was only partially attenuated c fos or unaffected  **zif268**  by  **NMDA**  receptor blockade.  Ref: 11102574 Brain Res, 2000
